# Prospective Evaluation of Risk Factors Responsible for Infection Following Retrograde Intrarenal Surgery

**DOI:** 10.7759/cureus.59420

**Published:** 2024-04-30

**Authors:** Kishan Raj K, Prashant Adiga K, Reshmina Chandni Clara D'souza, Nandakishore B, Rajani Upadhyaya

**Affiliations:** 1 Urology, Father Muller Medical College and Hospital, Mangalore, IND; 2 General Surgery, Father Muller Medical College and Hospital, Mangalore, IND; 3 Obstetrics and Gynaecology, Kasturba Medical College, Manipal, Udupi, IND

**Keywords:** diabetes mellitus, modified clavien classification system, fever, urinary tract infection, retrograde intrarenal surgery

## Abstract

Objective: The study aimed to identify the various risk factors for infective complications following retrograde intrarenal surgery (RIRS).

Materials and methods: The study was conducted over one year, and the incidence of infectious complications after RIRS was calculated. Patients were divided into two groups based on the presence and absence of infective complications and were compared in terms of preoperative and operative characteristics. The complications were assessed and graded according to the Modified Clavien classification system (MCCS). The Fisher's exact test, Student's t-test, and Mann-Whitney U test were used for univariate analysis. Multivariate logistic regression analysis was used to identify independent risk factors for postoperative urinary tract infection (UTI).

Results: Out of 165 patients in the study, 27 (16.7%) patients developed UTI within one month of undergoing RIRS. The most frequent complication was fever, which occurred in 13 (7.8%) patients. When stratified by MCCS, 13 were grade I, nine were grade II, four were grade III, and one was a grade IV complication. High stone burden, concomitant diabetes mellitus, and multiple renal stones were identified as substantial risk factors for postoperative UTI in univariate analysis. On multivariate analysis, preoperative UTI and prolonged operative time were found to have a significant association with postoperative UTI.

Conclusion: The present study demonstrated that preoperative UTI and prolonged operative time are independent factors responsible for postoperative UTI. Large stone burden, stone multiplicity, and diabetes mellitus contribute to a higher risk for UTI following RIRS.

## Introduction

There has been a drastic change in the management of renal stones over the last few decades. Percutaneous nephrolithotomy (PCNL) and retrograde intrarenal surgery (RIRS) are the most common endourological procedures for treating upper urinary tract stones [1]. Stone location, burden, and complexity are the deciding factors before treatment selection. PCNL has traditionally been the procedure of choice for stones >2 cm and complex renal stones [2]. Although PCNL offers a higher stone-free rate (SFR) compared to RIRS, it has its own set of complications like bleeding, colon injury, and sepsis. The Clinical Research Office of the Endourological Society (CROES) PCNL Global Study reported that 20.5% of its population developed complications following PCNL [3]. RIRS with flexible ureteroscope allows the removal of renal stones with minimal injury to renal parenchyma and has a decreased risk of bleeding. RIRS has fewer complications and near equivalent SFRs compared to conventional PCNL. RIRS is considered a first-line treatment option for renal stones <20 mm [4]. This has greatly helped in reducing the duration of hospital stays and patient morbidity. Recent technical refinements in RIRS, including a smaller caliber of scopes, better light transmission, and an expanded field of vision, have popularized its usage and broadened its indications in renal stone management [5].

Infective complications like fever and sepsis are potential complications of RIRS. Preoperative sterile urine culture and strict adherence to preoperative antimicrobial prophylaxis are crucial in decreasing the rate of infection post-RIRS. Despite adhering to strict protocols, infection remains a significant concern, with reported incident rates of 1.7%-18.8% [6]. 

We aim to conduct an in-depth study to evaluate the prevalence of infective complications after RIRS and identify risk factors responsible for postoperative infective complications following RIRS.

## Materials and methods

Study design and population

This prospective study was conducted on patients who were admitted for RIRS in the Department of Urology, Father Muller Medical College, Mangalore, India between December 2022 and November 2023. The study was commenced after obtaining approval from the Father Muller Institutional Ethics Committee (FMIEC/CCM/793/2022). The study was performed in accordance with the Declaration of Helsinki. Written informed consent was taken from all participants of the study.

All patients above 18 years with 10-35 mm renal stones were enrolled in the study. Patients with preoperative double-J (DJ) stents and those who had undergone renal stone-related procedures in the past were also included in the study. Pregnant women and patients with severe cardiac insufficiency and neurological disorders were excluded from the study. Patients with severe hemorrhagic disorders, uncontrolled urinary tract infection (UTI), and severe immune deficiency were not included in the study. We also excluded patients with long-term indwelling catheters and those who underwent bilateral RIRS.

The following data was collected from patients who were included in the study: age, sex, history of surgical treatment of renal stones, history of UTI in the preceding month, and specific comorbidities (diabetes mellitus, chronic kidney disease, coronary artery disease, hypertension, and hyperlipidemia).

All patients underwent computed tomography (CT) urogram to look for stone size, density, multiplicity, and anatomical abnormalities. Stone size was calculated as the maximum length of the stone, and in multiple stones, the total of the maximum lengths was taken into consideration. Patients with contraindications to intravenous contrast underwent a noncontrast CT scan. Urine culture was mandatorily performed for each patient. Patients with positive urine cultures and those with preoperative UTIs were treated with culture-specific antibiotics for one week. RIRS was performed only after obtaining sterile urine culture. Based on the hospital protocol, every patient received a single dose of injectable antibiotic (combination of cefoperazone with 1.5 g sulbactam) 30 minutes before surgery.

Surgical technique

After anesthesia induction, patients were placed in the low lithotomy position. All procedures were performed by two experienced surgeons with experience of more than 100 cases each. A preliminary cystoscopy was done, and any preplaced DJ stent was removed. A 7-Fr semirigid ureteroscope (Karl Storz, Germany) was used to inspect the ureter and check for compliance before insertion of ureteric access sheath (UAS) (Cook Medical, Bloomington, IN). A 7.5-Fr flexible digital ureterorenoscope (Biorad Medisys, India) was used for lithotripsy during RIRS. When the insertion of the UAS was difficult, a flexible ureterorenoscope was advanced directly into the kidney under fluoroscopic control across a guidewire. If any difficulty was encountered during the sheathless insertion of a flexible ureterorenoscope, a DJ stent was inserted, and RIRS was postponed for two weeks. Renal stones were fragmented with a 30-watt Holonium laser (Quanta, Italy) using 200-μm laser fiber. Irrigation fluid was placed at 60 cm height, and a manual irrigation pump was used to improve visualization during surgery. DJ stent was routinely placed at the end of the procedure. Patients were monitored postoperatively for vital signs. X-ray kidney, ureter, and bladder (KUB) was routinely performed on postoperative day one to look for the position of the DJ stent and the presence of residual stones. Patients were followed up after discharge on one week and four weeks post-surgery.

Definitions

Fever was defined as a temperature over 38°C that persisted for 48 h. Systemic inflammatory response syndrome (SIRS) was defined by two or more of the following criteria: (1) body temperature less than 36°C or greater than 38°C, (2) heart rate greater than 90 beats per minute, (3) respiratory rate greater than 20 breaths per minute, and (4) white cell count > 12,000/mm^3^ or <4000/mm^3^. For sepsis to be diagnosed, patients needed to have two SIRS criteria and confirmation of infection. Severe sepsis was defined as sepsis with signs of end-organ dysfunction. Operative time was calculated from the start of the semirigid ureteroscopy to the end of the procedure. SFR was defined as the absence of residual fragments larger than 4 mm one month after the procedure.

Outcome measures

Infectious complications were said to be present when the patient developed a fever of more than 38°C, SIRS, or sepsis within four weeks of surgery. The Modified Clavien Classification System (MCCS) was utilized to evaluate postoperative complications of RIRS. When patients had more than one complication, only the highest score was included in the final analysis. A noncontrast CT scan was done at four weeks to look for residual stones. The primary endpoint of this study was to measure the incidence of postoperative infectious complications. Preoperative and intraoperative risk factors that might have contributed to postoperative infection were analyzed.

Statistical analysis

The descriptive analysis was done using mean or median with standard deviation (SD) or interquartile range (IQR) for quantitative variables. Categorical variables were presented in frequencies along with respective percentages. After ascertaining the distribution of data (whether satisfying normality or not), the statistical comparisons for quantitative variables were done using the student's t-test or Mann-Whitney U test. The chi-square test was used to assess categorical variables. Multivariate logistic regression analysis was used to identify independent risk factors for postoperative UTI. All statistical analysis was performed using SPSS version 23 (IBM Corp., Armonk, NY). P-value < 0.05 was considered statistically significant.

## Results

The study included 165 patients who satisfied the strict inclusion criteria. Demographic details and baseline characteristics of patients are depicted in Table 1. About 116 (70.3%) of the total study participants were male. The median age of the study population was 52 years (IQR: 39-59). Comorbidities that were assessed included diabetes mellitus, hyperlipidemia, chronic kidney disease, and hypertension, which were present in 36.9%, 35.7%, 20%, and 47.3% of the patients, respectively. About 24 (14.5%) patients had preoperative UTIs, which were treated with culture-specific antibiotics before RIRS, and 44.8% of the patient population had undergone urinary stone-related procedures previously.

**Table 1:** Baseline characteristics of patients undergoing RIRS RIRS: Retrograde intrarenal surgery; URSL: Ureteroscopic lithotripsy; PCNL: Percutaneous nephrolithotomy; ESWL: Extracorporeal shock wave lithotripsy; UTI: Urinary tract infection; SD: Standard deviation; IQR: Interquartile range.

Variables	Overall (%)	Non-infectious group (%)	Infectious group (%)	P-value
Number of patients	165	138 (83.6%)	27 (16.4%)	
Age in years (median, IQR)	52 (39-59)	51 (39-59)	52 (43-59)	0.62
Female gender	49 (29.7%)	37 (75.6%)	12 (24.4%)	0.07
Diabetes mellitus	61 (36.9%)	45 (32.6%)	16 (59.2%)	0.01
Chronic kidney disease	33 (20%)	24 (17.4%)	9 (33.3%)	0.06
Hypertension	78 (47.3%)	63 (45.6%)	15 (55.5%)	0.35
Coronary artery disease	29 (17.6%)	26 (18.8%)	3 (11.1%)	0.34
Hyperlipidemia	59 (35.7%)	49 (35.5%)	10 (37.0%)	0.88
Preoperative UTI	24 (14.5%)	12 (8.7%)	12 (44.4%)	<0.01
History of previous stone treatment	74 (44.8%)	59 (42.7%)	15 (55.5%)	0.22
URSL	47	38	9	
PCNL	14	10	4	
ESWL	9	8	1	
Open stone surgery	4	3	1	
Anatomical abnormality	19 (11.5%)	16 (11.6%)	3 (11.1%)	0.94
Use of ureteric access sheath	131 (79.4%)	111 (80.4%)	20 (74.1%)	0.46
Multiple renal stones	67 (40.6%)	44 (31.9%)	23 (85.2%)	<0.01
Stone-free rate	133 (80.6%)	114 (82.6%)	19 (70.4%)	0.14
Duration of surgery in minutes (mean ± SD)	44.9 (±20.70)	40.4 (±18.1)	68.3 (±17.0)	<0.01
Hounsfield unit of stone (mean ± SD)	1031 (±352)	1024 (±339)	1064 (±419)	0.59
Stone size in mm (mean ± SD)	19.24 (±6.7)	17.7 (±5.8)	27.0 (±5.4)	<0.01

Mean stone size and surgery duration were 19.24 mm (±6.7) and 44.9 min (±20.4), respectively. Moreover, multiple renal stones were present in 67 (40.6%) patients. UAS was placed in 131 (79.4%) patients, and complete stone clearance was achieved in 133 (80.6%) patients. Furthermore, 19 of the 168 study participants had renal anatomical abnormalities. These included nine patients with malrotated kidneys, three patients with calyceal diverticula, four patients with horseshoe kidneys, and three patients with pelvic kidneys. In total, 27 patients (16.4%) developed postoperative UTI within one month of RIRS.

Infectious complications were classified by MCCS as depicted in Table 2. Thirteen (7.8%) patients developed a fever, which was classified as a grade I complication. SIRS and sepsis occurred in six (3.6%) and three (1.8%) patients, respectively. Four patients (2.4%) with grade III complications required operative intervention. Three patients required DJ stent repositioning for obstructive pyelonephritis, and one patient required pigtail insertion for perinephric collection. One (0.6%) patient was readmitted to the intensive care unit for severe sepsis, which was a grade IV complication.

**Table 2:** Infective complications according to MCCS MCCS: Modified Clavien classification system; SIRS: Systemic inflammatory response syndrome.

Grade	Complication	Total number of patients (%)
I	Fever	13 (7.8%)
II	SIRS	6 (3.6%)
	Sepsis	3 (1.8%)
III	Obstructive pyelonephritis	4 (2.4%)
IV	Severe sepsis	1 (0.6%)

Univariate analysis (Table 1) identified diabetes mellitus (p = 0.01) and preoperative UTI (<0.01) as strong predictors of postoperative UTI. Cases with longer operative duration had a statistically higher risk of postoperative UTI (p < 0.01). Large stone size (p < 0.01) and stone multiplicity (p < 0.01) were strong preoperative predictors for postoperative UTI. Variables that showed significant statistical association in univariate analysis were analyzed by multivariable logistic regression analysis (Table 3). Preoperative UTI (p = 0.02, OR 4.412, 95% CI = 1.263-15.412) and longer operative duration (p = 0.02, OR 1.079, 95% CI = 1.011-1.152) were the two independent predictors of postoperative UTI in multivariable logistic regression analysis.

**Table 3:** Multivariable logistic regression analysis of variables associated with infective complications OR: Odds ratio; CI: Confidence interval.

Factors	OR (95% CI)	P-value
Diabetes mellitus	2.685 (0.838-8.601)	0.09
Multiple renal stones	3.022 (0.729-12.535)	0.13
Preoperative UTI	4.412 (1.263-15.412)	0.02
Stone size	1.094 (0.945-1.266)	0.23
Duration of surgery	1.079 (1.011-1.152)	0.02

## Discussion

The prevalence of urolithiasis has been increasing steadily over the past few decades and is the third most common pathological condition affecting the urinary tract. When classified based on the location, upper urinary tract stones are seen in the majority of patients. RIRS is increasingly becoming popular among urologists and patients because of its non-invasive nature, shorter hospitalization time, and lower complication rates. Studies have shown excellent SFR comparable with PCNL and a lower risk of renal parenchymal damage and bleeding complications [7]. Bozkurt et al. compared PCNL and RIRS success rates for stones between 1.5 and 2 cm and reported an SFR of 92.8% for PCNL and 89.2% for RIRS [8]. Recent advances in RIRS technology, particularly laser lithotripsy, have expanded indications of RIRS. RIRS can easily be staged and is being increasingly used for stones >2 cm. Karakoç et al. reported SFR as high as 87.7% after two sessions of RIRS compared to 66.6% for a single session of RIRS [9].

However, infection post-RIRS remains a worrisome complication. These include acute UTI, SIRS, and sepsis [10]. There is colonization of bacteria inside stones, and they are released along with endotoxins during stone fragmentation, thereby triggering a systemic inflammatory response. Incidence of infection post-RIRS ranged from 1.7% to 18.8% in a multitude of studies [6]. This varied incidence in published literature is not only due to underreporting but also due to non-standardized classification of infectious complications. Corrales et al. conducted a systematic review and found that the rate of sepsis in RIRS ranged from 0.5% to 11.1% [10]. Third International Consensus Definitions for Sepsis and Septic Shock have defined sepsis as life-threatening organ dysfunction caused by a dysregulated host response to infection [11]. SIRS and sepsis can progress to multiple organ dysfunction syndrome (MODS), with mortality rates as high as 20% [12]. We observed a relatively high rate of postoperative UTI, with 16.4% of our patients reporting infectious complications. This could be because a majority of our patients had larger stone burdens and longer operative times.

MCCS is a standardized and validated classification system for reporting infectious complications after surgery. Standardized reporting helps us compare complication rates and their severity among different centers. The majority of complications were grade I in nature (48.1%), with postoperative fever being the most common complication occurring in 7.8% of patients. This is consistent with a study by Berardinelli et al., where fever was the most common complication occurring in 4.4% of patients [13].

Preoperative UTI is a strong predisposing factor for postoperative UTI. In our study, 12 out of 24 (50%) patients with positive preoperative positive urine culture had fever. Patients with UTI in the preceding month had a 4.4 times higher risk for postoperative UTI (95% CI: 1.263-15.412, p = 0.02). Pérez et al., in their study on 236 patients undergoing RIRS, concluded that a history of treatment for urinary sepsis was a strong risk factor for postoperative UTI [14]. Similar results were reported by Baboudjian et al., who found that UTIs in the preceding six months increased infection risk by 2.3 times [15]. It is hypothesized that biofilm coating in long-standing stones makes it difficult for antibiotics to kill bacteria. Preoperative infection also carries a high risk for stent colonization [16]. Furthermore, urine culture does not accurately reflect causative organisms as renal pelvic or stone cultures. Margel et al. highlighted this, finding only 30% sensitivity of urine culture to predict stone colonization in patients undergoing PCNL [17]. Nevertheless, prompt evaluation and treatment of preoperative UTIs based on urine culture and susceptibility testing are imperative to prevent postoperative infections.

Studies have reported prolonged operative time as a risk factor for postoperative fever. It is advisable to limit operative time below 60 minutes and stage the procedure if necessary. In a study by Ozgor et al., infection risk increased by 2.36-fold when operative time crossed 60 min [18]. Sugihara et al. constructed a nomogram to predict adverse events after ureteroscopic lithotripsy and found a positive correlation between operative time and postoperative urinary infection [19]. Dissemination of bacterial endotoxins occurs during stone fragmentation. Prolonged surgery likely causes increased pyelolymphatic and pyelovenous backflow, thereby augmenting infection risk. In multivariate analysis, prolonged operative time increased infection risk by 1.079 times (95% CI: 1.011-1.152, p = 0.02).

Xu et al. concluded that patients with higher stone burden have an increased infection risk [20]. Larger stones harbor higher bacterial load and require more time for fragmentation. Although stone size positively influenced infection risk in univariate analysis (p < 0.01), we could not find an independent association between stone size and infection risk in multivariate analysis.

Stone size is closely related to SFR, with large-diameter stones having an increased risk for residual stones. A recent study has shown incomplete stone clearance as a risk factor for postoperative UTI [13]. However, this was not our experience, and we did not find a strong association between SFR and postoperative UTI (p = 0.14).

In our analysis, we found that patients with multiple stones undergoing RIRS had an increased risk for postoperative UTI (p < 0.01). In a large prospective study by Bas et al., stone size, stone number, and the presence of congenital abnormalities were factors that were shown to influence infection risk in RIRS [21]. We believe that a strong correlation exists between stone multiplicity and total stone burden. This may be why we did not find stone multiplicity as an independent predictor for postoperative infection.

Our study demonstrated a strong association between diabetes mellitus and postoperative UTI. About 59.2% of patients with diabetes mellitus developed UTI compared to 32.6% who did not have diabetes mellitus (p = 0.01). In a prospective study by Li et al., preoperative diabetes mellitus was found to be a strong predictor of postoperative urinary sepsis [22]. Patients with long-standing diabetes have depressed phagocyte and granulocyte function with impaired immune response [23]. Microvascular disease in diabetic patients drastically reduces oxygen delivery to peripheral tissues. Additionally, cystopathy occurring in long-standing diabetes mellitus causes impaired bladder emptying, making patients more prone to UTIs. American Urology Association guidelines recommend deferring RIRS if preoperative blood sugar levels exceed 400 mg/dl [24].

Our analysis did not find renal anatomical abnormality as a risk factor for postoperative UTI (p = 0.94). Partial pelvic ureteric junction obstruction is common in analogous kidneys. Increased intrapelvic pressure can increase infectious risk during RIRS in these kidneys. Ozgor et al. found the presence of renal anatomical abnormality to be a strong risk factor for postoperative UTI [18]. The low number of cases with anomalous kidneys in our study precluded us from finding a significant association.

Zhong et al. have demonstrated that intrarenal pressures > 30 mm increase the likelihood of postoperative urinary sepsis [25]. High-volume irrigation flow and the absence of UAS have a summative effect on intrarenal pressure. UAS has been shown to decrease intrarenal pressure by as much as 75% [26]. UAS usage facilitates repeated ureteroscope manipulation, reduces trauma to the ureter, and shortens operative time. Most importantly, UAS reduces intrapelvic pressure and, thereby, irrigation fluid intravasation. Despite the above advantages, we found no significant association between UAS usage and postoperative fever (p = 0.15). Gravity irrigation is the most effective method for maintaining low irrigation pressures during RIRS [27]. Xu et al. recommended an irrigation flow rate below 25 ml/min during RIRS [20]. It is thought that this flow rate precludes an excessive increase in intrarenal pressure. Although hand syringe irrigation and automated pumps can improve visualization, low flow rates are best achieved with gravity irrigation [27].

Struvite stones form due to infection with urea-splitting organisms like *Proteus*, *Klebsiella*, and *Staphylococcus *species [28]. These bacteria produce urease that causes the breakdown of urea into ammonia and carbon dioxide. High levels of urea create an alkaline environment that drives stone growth. Lithotripsy of struvite stones is fraught with a higher risk of UTI. Peng et al. conducted a study on 1493 patients and found infection stones to be a substantial risk factor for postoperative UTI [29]. Unfortunately, we did not include data on stone composition in our study.

Berardinelli et al. found increased rates of UTI in patients who had undergone past surgery for renal stones [13]. We did not find previous urinary tract surgeries to be a risk factor for postoperative UTI.

This study had certain limitations. Our study did not investigate the possible effect of higher renal pelvic pressure on postoperative infectious complications. Renal pelvic urine culture and stone culture are important determinants that were not investigated in the present study. Lastly, we should have included stone composition analysis in our study. A multicenter study with a large sample size is needed to validate our results.

The strengths of our study include standardized criteria for defining infectious complications based on MCCS. This helps to reduce confounding and allows adequate comparision of post-surgical infection between different centers. Additionally, all the procedures were performed by two experienced surgeons, which is a major strength of our study.

## Conclusions

Based on the present study, we could conclude that preoperative UTI and prolonged operative time are independent factors responsible for postoperative UTI. Large stone burden, stone multiplicity, and diabetes mellitus carry a higher risk for postoperative UTI. We recommend limiting operative time and staging the procedure in patients with large stone burden and multiple stones. Recognition of risk factors like preoperative UTI and diabetes mellitus is paramount to prevent infectious complications after RIRS.
